# EMMPRIN/CD147 plays a detrimental role in clinical and experimental ischemic stroke

**DOI:** 10.18632/aging.102935

**Published:** 2020-03-19

**Authors:** Anthony Patrizz, Sarah J. Doran, Anjali Chauhan, Hilda Ahnstedt, Meaghan Roy-O’Reilly, Yun-Ju Lai, Gillian Weston, Sami Tarabishy, Anita R. Patel, Rajkumar Verma, Ilene Staff, Julia K. Kofler, Jun Li, Fudong Liu, Rodney M. Ritzel, Louise D. McCullough

**Affiliations:** 1 The University of Texas Health Science Center at Houston and the McGovern Medical School, Houston, TX 77030, USA; 2 Department of Neuroscience, University of Connecticut Health Center, Farmington, CT 06030, USA; 3 The Stroke Center at Hartford Hospital, Hartford, CT 06102, USA; 4 Department of Anesthesiology, Center for Shock, Trauma, and Anesthesiology Research, University of Maryland School of Medicine, Baltimore, MD 21201, USA; 5 Department of Pathology, University of Pittsburgh School of Medicine, Pittsburgh, PA 15261, USA

**Keywords:** stroke, middle cerebral artery occlusion, CD147, EMMPRIN, aging

## Abstract

Background: Ischemic stroke is a devastating disease, often resulting in death or permanent neurological deficits. EMMPRIN/CD147 is a plasma membrane protein that induces the production of matrix metalloproteinases (MMPs), which contribute to secondary damage after stroke by disrupting the blood brain barrier (BBB) and facilitating peripheral leukocyte infiltration into the brain.

Results: CD147 surface expression increased significantly after stroke on infiltrating leukocytes, astrocytes and endothelial cells, but not on resident microglia. Inhibition of CD147 reduced MMP levels, decreased ischemic damage, and improved functional, cognitive and histological outcomes after experimental ischemic stroke in both young and aged mice. In stroke patients, high levels of serum CD147 24 hours after stroke predicted poor functional outcome at 12 months. Brain CD147 levels were correlated with MMP-9 and secondary hemorrhage in post-mortem samples from stroke patients.

Conclusions: Acute inhibition of CD147 decreases levels of MMP-9, limits tissue loss, and improves long-term cognitive outcomes following experimental stroke in aged mice. High serum CD147 correlates with poor outcomes in stroke patients. This study identifies CD147 as a novel, clinically relevant target in ischemic stroke.

## INTRODUCTION

Ischemic stroke is a leading cause of death and disability in the United States [1]. Post-stroke inflammation contributes to blood brain barrier (BBB) breakdown via the up-regulation of inflammatory chemokines, cytokines, adhesion molecules and matrix metalloproteinases (MMPs), facilitating entry of peripheral leukocytes into the brain [2–4]. Leukocyte trafficking into the brain after stroke contributes to secondary injury via several mechanisms, including the release of pro-inflammatory cytokines [5, 6]. The post-stroke inflammatory response is triggered immediately after injury, primarily by microglia [7], but peaks as late as 72 hours after injury, making it an attractive therapeutic target [8, 9].

Many cells in the injured brain, including neurons, endothelial cells, platelets, astrocytes, neutrophils and monocytes/macrophages, secrete MMP-9 [3, 10, 11]. Secreted MMP-9 degrades extracellular matrix proteins, increasing BBB permeability and hemorrhagic transformation in the brain after ischemic stroke [4, 12]. Extracellular matrix metalloproteinase inducer (EMMPRIN/CD147) is a cell surface glycoprotein that induces the production of MMPs, including MMP-9 [13]. Due to its ability to induce MMP activity, CD147 promotes leukocyte extravasation through blood vessels [14, 15]. Previous studies have shown that CD147 is up-regulated after myocardial infarction and in chronic inflammatory conditions, including atherosclerosis and multiple sclerosis [16]. Following transient middle cerebral artery occlusion (MCAO), micro vessel expression of CD147 as well as MMP-9 activity is increased [17].

Both clinically and experimentally, increased plasma levels of MMP-9 correlate with a higher risk of hemorrhagic transformation [18, 19], and poor outcomes [20, 21]. Thus, MMP-9 inhibition is thought to be a potential target for stroke therapy. Several different treatment approaches have been attempted, yet due to the lack of MMP inhibitor specificity, no MMP-9 blocking treatments have been developed [4]. Inhibition of CD147 using a selective blocking antibody may be an effective alternative approach to limiting MMP activity after ischemic stroke. A recent study reported that inhibition of CD147 improves short-term stroke outcomes (72 hours) in young animals via the suppression of inflammation and endothelial dysfunction [17]. However, if acute inhibition of CD147 is beneficial or detrimental to long-term stroke outcomes is unknown. Furthermore, as more than 80% of strokes occur in individuals over the age of 65, [22] and hemorrhagic transformation is more severe with advanced age, the effects of CD147 inhibition in aged animals requires evaluation.

We hypothesized that inhibition of CD147 would decrease levels of MMP-9, reduce histological damage, and enhance long-term functional and cognitive outcomes in aged animals, which have a greater risk of post-stroke hemorrhagic transformation. In addition, we evaluated the clinical relevance of CD147 as a potential therapeutic target. Serum CD147 levels in patients 24 hours after acute ischemic stroke were measured and correlated to functional outcomes (Modified Rankin score) one year post-stroke. CD147 expression was also examined in post-mortem brain tissue from stroke patients and age-matched controls.

## RESULTS

### CD147 co-localizes with astrocytes and endothelial cells

**Figure 1:**
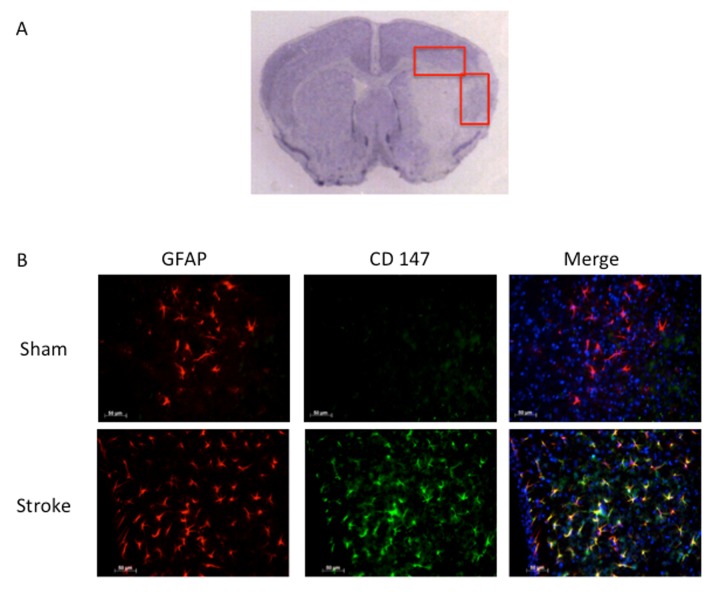
**GFAP positive astrocytes co-localize with CD147.** (**A**) Representative image of cresyl violet stained brain slice showing peri-infarct regions where imaging was performed. (**B**) Representative 20x IHC images of sham and stroke brain slices. CD147 co-localizes with GFAP positive astrocytes in the peri-infarct region. Pearson’s correlation coefficient <0.0001, SH 0.14±0.02 vs. ST 0.73±0.03, n=6.

**Figure 2:**
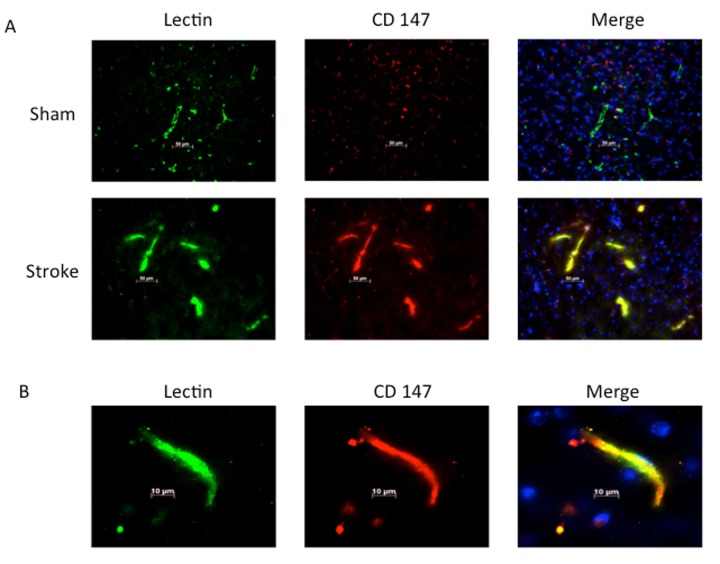
**Lectin positive endothelial cells co-localize with CD147.** (**A**) Representative 20x IHC images of sham and stroke brain slices indicating CD147 co-localizes with lectin positive endothelial cells. Pearson’s correlation coefficient <0.0001, SH 0.29±0.01 vs. ST 0.748±0.03, n=3 and 5 respectively. (**B**) 63x high magnification image of lectin with co-localization with CD 147 in stroke brain.

Consistent with previous reports, CD147 increased in the peri-infarct region (area shown in Figure 1A) by immunohistochemistry (IHC) [16]. Co-localization of CD147 with glial fibrillary acidic protein (GFAP)-positive astrocytes was seen in stroke mice but not in sham mice at 72h (Pearson’s correlation coefficient <0.0001, sham (SH) 0.14±0.02 vs. stroke (ST) 0.73±0.03, n=6, Figure 1B). This increase in CD147 expression was observed specifically in astrocytes within the penumbra. CD147 also co-localized with cerebral endothelial cells after stroke, as visualized by lectin staining (Pearson’s correlation coefficient <0.0001, SH 0.29±0.01 vs. ST 0.74±0.03, n=3 and 5 respectively, Figure 2). Our current findings confirm previous reports of co-localization of CD147 both with endothelial cells and GFAP in permanent focal ischemia [16].

### Brain-infiltrating monocytes up-regulate CD147 expression:

**Figure 3:**
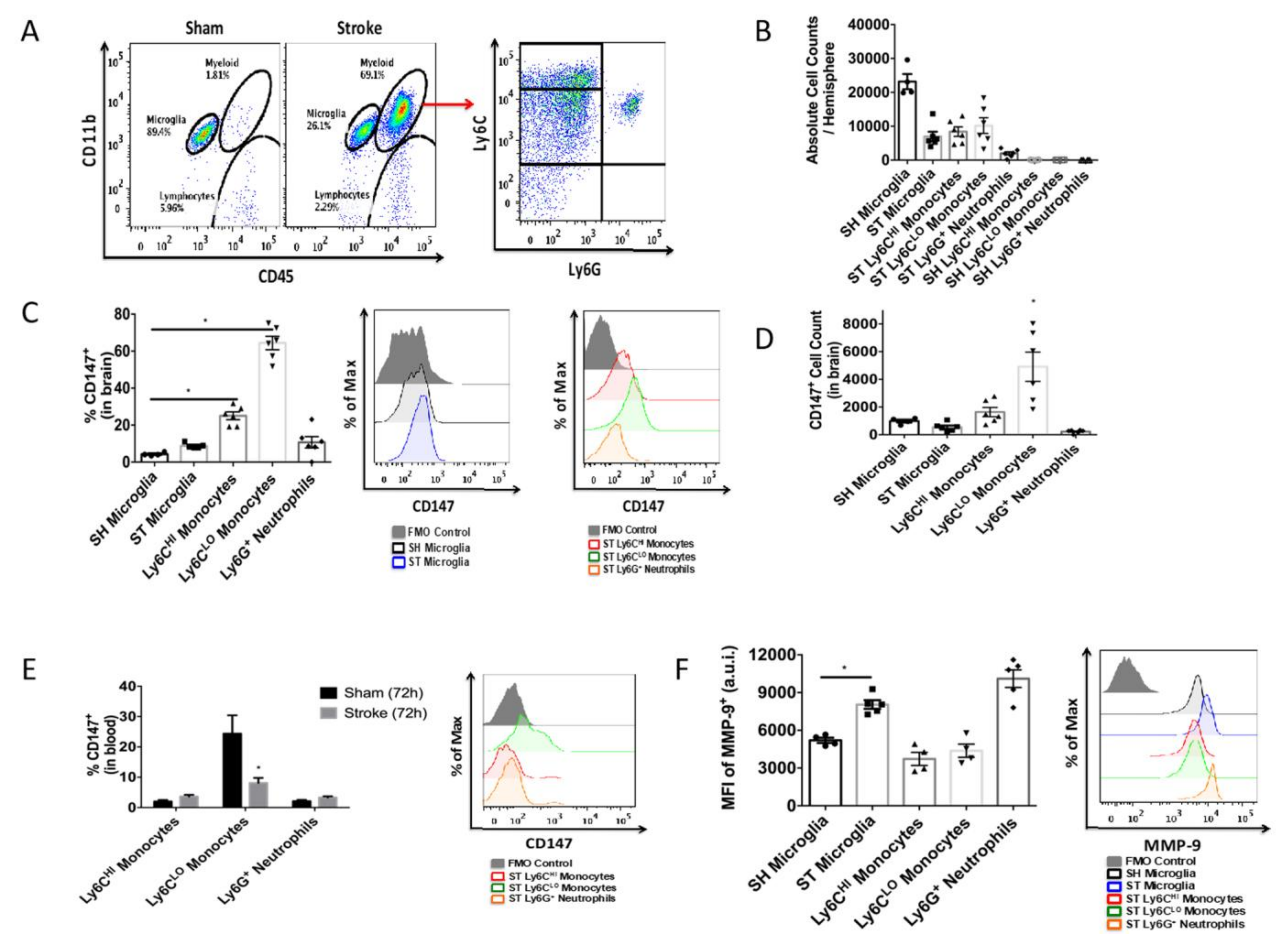
**CD147 is expressed on brain infiltrating monocytes.** (**A**) Representative dot plot of sham and stroke (72hr) brain shows microglia and infiltrating myeloid cells. Representative dot plot of 72hr stroke brain, gated on myeloid cells, shows Ly6C^hi^ monocytes, Ly6C^lo^ monocytes, and Ly6G neutrophils. (**B**) Absolute cell counts at 72hr in stroke brain (and sham microglia). (**C**) Quantified % CD147 positive cells in brain. Representative histogram showing expression of CD147 on microglia (sham and stroke; gray= microglia-specific FMO). Representative histogram showing expression of CD147 on brain-infiltrating myeloid cells after stroke. (**D**) Quantified CD147^+^ cell counts from brain, n=6. (**E**) Quantified %CD147 positive blood cells. Representative histogram showing expression of CD147 on blood cells after stroke. (**F**) Quantified expression level of MMP-9 on brain-infiltrating myeloid cells (and sham microglia). Representative histogram showing expression of MMP-9 on brain-infiltrating myeloid cells (and sham microglia).

To quantitatively assess CD147 expression, flow cytometry of brain tissue was performed using the gating strategy depicted in Figure 3A. Peripheral myeloid cells, including infiltrating Ly6C^hi^ monocytes, Ly6C^lo^ monocytes, and Ly6G^+^ neutrophils were found to increase significantly in the brain following ischemic stroke in a manner that was inversely proportional to microglia number (Figure 3B), in agreement with previous work by our laboratory and others [7].

At 72 hours after injury, CD147 surface expression was significantly increased in peripheral monocyte populations found in the ischemic brain when compared to sham brain or their circulating counterparts (Figure 3C, 3D). Importantly, Ly6C^lo^ monocytes within the stroke brain expressed CD147 at a significantly higher level than activated resident microglia at 72 hours after stroke (Figure 3C, p=0.001, n=6). CD147 expression was found on nearly 60% of brain-infiltrating Ly6C^lo^ monocytes, compared to just 10% of the monocytes circulating in the blood after stroke, whereas Ly6G^+^ neutrophils showed relatively little expression in brain or blood (Figure 3C, 3D and 3E). Interestingly, despite the lack of CD147 up-regulation on resident microglia, there was a significant increase in the target of CD147, MMP-9, in microglia from stroke mice compared to sham (p=0.001, n=7, Figure 3F). Ly6G^+^ neutrophils demonstrated the highest production level of MMP-9 in the ischemic brain at this time point, as seen in other studies [23, 24].

### A CD147 blocking antibody reduced MMP-9 levels and improved histological outcomes 72 hours after stroke:

**Figure 4:**
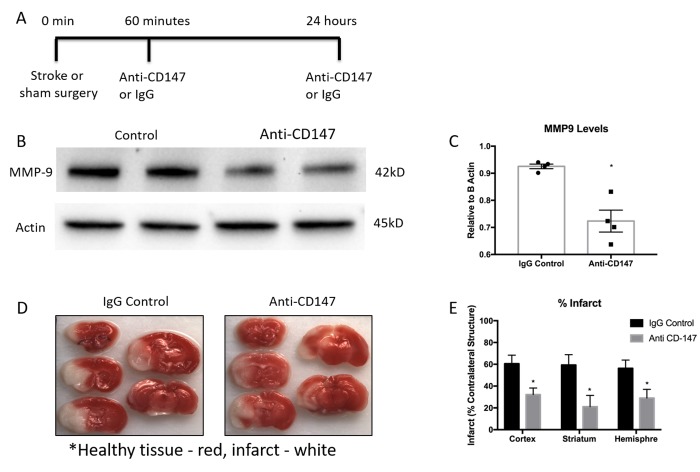
**Matrix metalloproteinase-9 (MMP-9) protein concentrations and infarct volumes decrease following anti-CD147 antibody administration.** (**A**) Timeline depicting the dosing strategy of CD147. (**B**) Representative western blots of MMP-9 protein concentrations in 72-hour post stroke mice following IgG control or anti-CD147 antibody administration, quantified in (**C**) p<0.01, n=4, * indicates statistical significance. (**D**) Representative TTC images of 72 hour post stroke brain slices of IgG control or ant-CD147 antibody. Red color details healthy brain tissue while white is indicative of infarcted tissue. (**E**) Quantification of percent infarct volume of cortex, striatum or total hemisphere relative to contralateral regions, p<0.05 for each region, n=4.

As previous reports have shown an increase in CD147 expression and MMP-9 activity in the ischemic brain, we utilized a CD147 blocking antibody in an attempt to reduce levels of MMP-9. Tail vein administration of CD147 blocking antibody, following the dosing timeline depicted in Figure 4A, reduced protein expression of MMP-9 when measured 72 hours after stroke (IgG control 0.925±0.008 vs. CD147 antibody 0.723±0.04 relative to actin, p<0.01, n=4, Figure 4B and quantified Figure 4C).

CD147 blocking antibody administration reduced 72-hour infarct volumes in the cortex (IgG 60.56±7.81 vs. CD147 Ab 32.23±6.0, p<0.05), striatum (IgG 59. 38±9.4 vs. CD147 Ab 21.14±10.3, p<0.05) and hemisphere (IgG 56.26±7.6 vs. CD147 Ab 29.04±8.0, n =4, p<0.05) of stroke animals compared to those that received IgG control, consistent with prior studies [17]. Representative TTC images are shown in Figure 4D, quantified data are shown in Figure 4E.

### CD147 blocking antibody improves histological and cognitive outcomes in young and aged mice following MCAO

**Figure 5:**
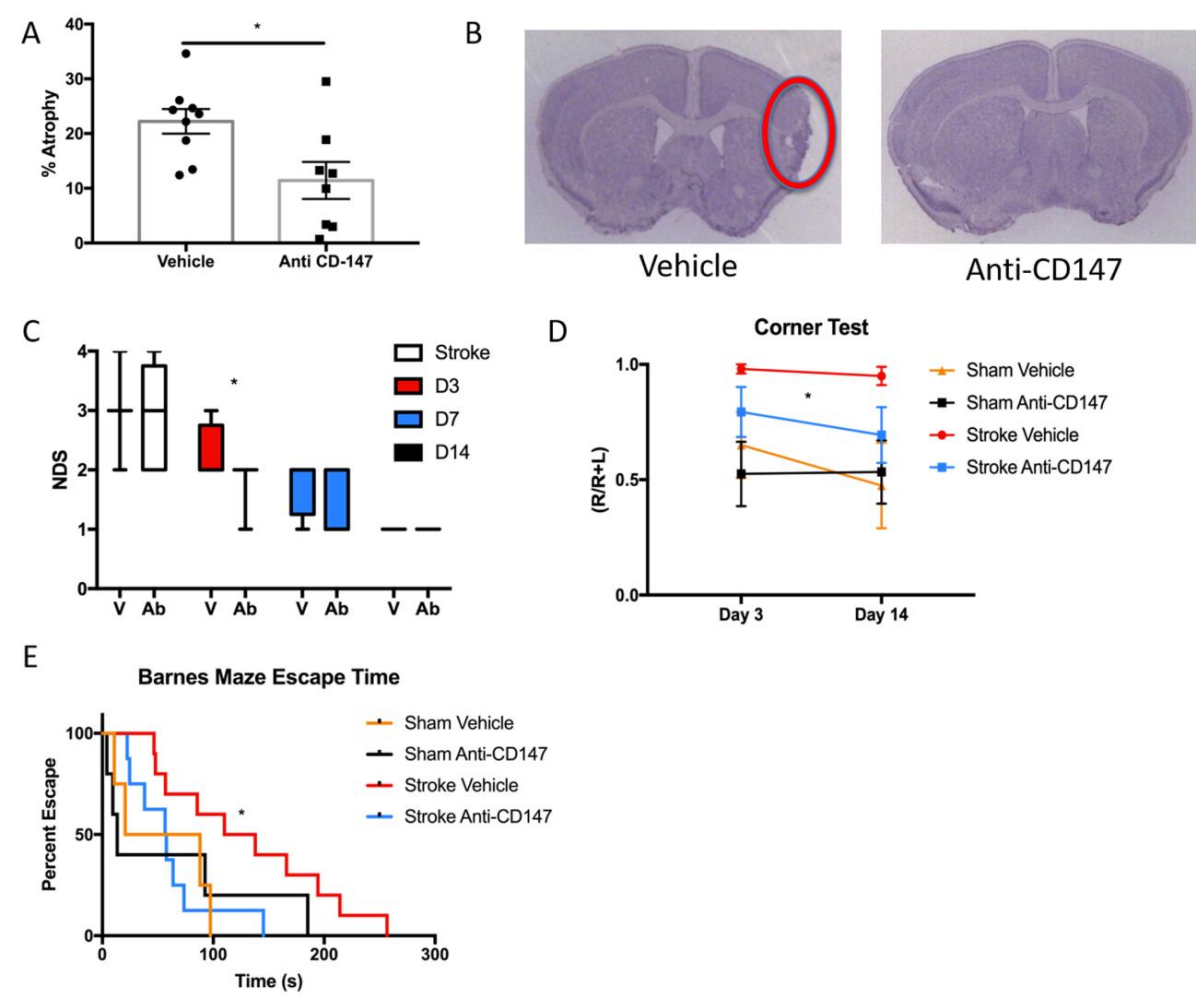
**Histological and Cognitive outcomes are improved following anti-CD147 antibody administration.** (**A**) Quantification of percent atrophy of brain slices 14 days following MCAO, p<0.05, n=9. (**B**) Representative cresyl violet images of IgG or anti-CD147 antibody administered slices, region of interest highlighted by red oval. (**C**) Neurological deficit scores were obtained at four time points over 14 days beginning with one-hour post onset of MCAO. Day 3 post MCAO anti-CD147 administered mice displayed improved NDS, p<0.05. By day 14 NDS were the same between both groups. (**D**) Corner testing revealed an effect of antibody treatment across two time points; day 3 and day 14, p<0.05. (**E**) Barnes maze testing highlighted improvement in spatial learning and memory in antibody treated mice compared to IgG control on day 14, p=0.03.

To determine the long-term implications of acutely blocking CD147, we studied histological and behavioral outcomes over a 14-day period following ischemic stroke. CD147 blockade reduced the volume of tissue atrophy compared to IgG treated stroke mice (IgG 22.23±2.25 vs. CD147 Ab 11.43±3.38%, p<0.05, n=9/grp, Figure 5A). Representative cresyl violet (CV) images of CD147 and IgG treated mice are shown in Figure 5B. Antibody treated mice also displayed lower neurological deficit scores (NDS) 72-hour post stroke compared to vehicle treated strokes (p=0.05, generalized linear mixed model with Bonferroni correction, Figure 5C). A main effect of antibody treatment was observed on the corner test (p<0.05, 2-way ANOVA, Figure 5D) with treated stroke mice displaying decreased sensory-motor impairment. Spatial memory and learning tested on the Barnes maze was preserved in stroke mice who received anti-CD147 antibody compared to isotype control treated mice, as evidenced by a shorter time to find the escape hole on day 14 (p=0.03, HR=0.309, Cox model with Tukey’s multiple comparisons test, Figure 5E). No difference was noted between sham groups.

**Figure 6:**
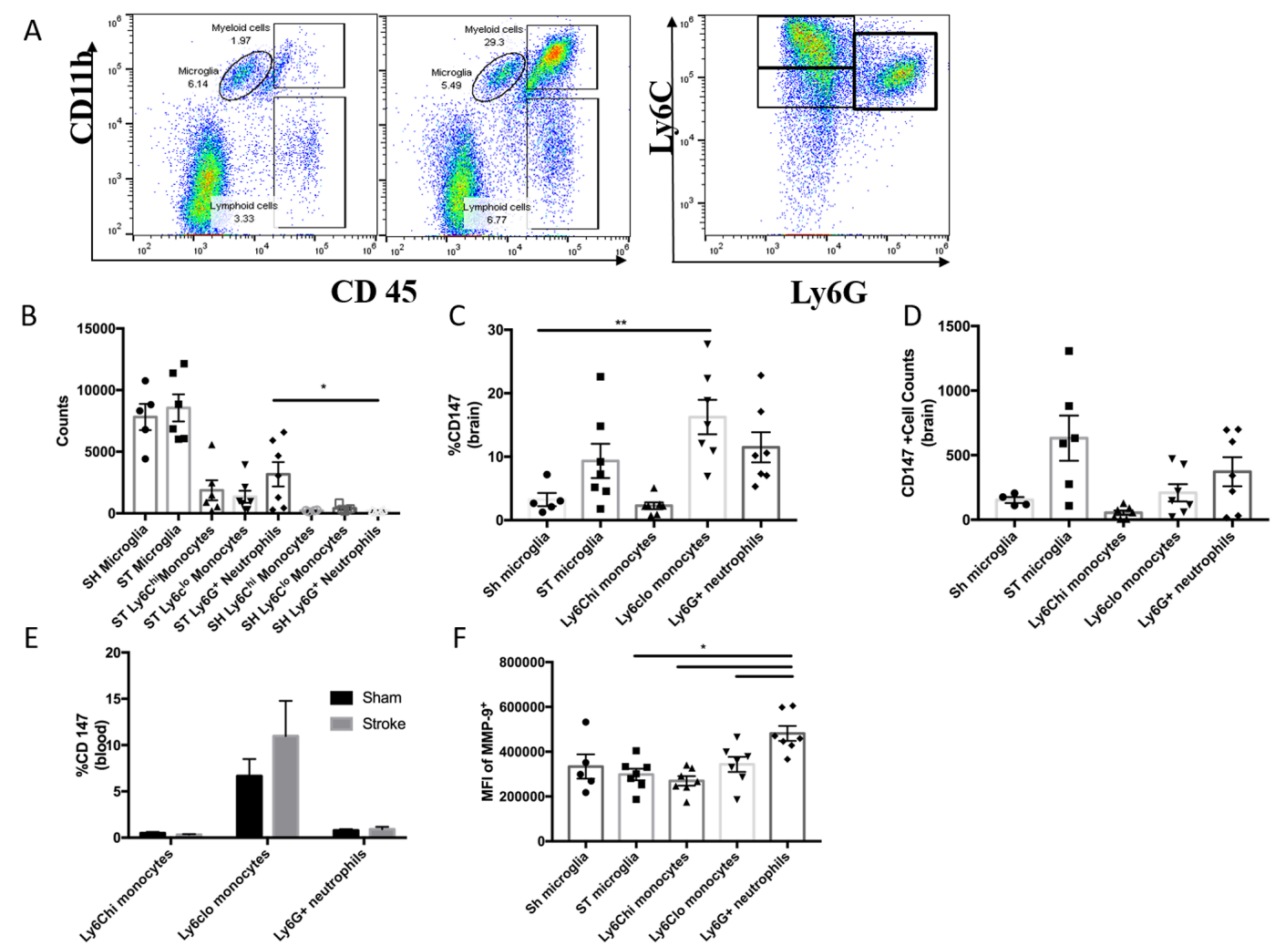
**CD147 expression in the aged brain.** (**A**) Representative dot plot of aged sham and stroke (72hr) brain; shows microglia and infiltrating myeloid cells. Representative dot plot of 72hr stroke brain; gated on myeloid cells, shows Ly6C^hi^ monocytes, Ly6C^lo^ monocytes, and Ly6G neutrophils. (**B**) Quantified counts at 72hr in stroke brain and sham microglia. (**C**) Quantified % CD147 positive cells in brain. (**D**) Absolute counts of CD147^+^ cells in brain, n=6. (**E**) Quantified %CD147 postive cells from blood. (**F**) Quantified expression level of MMP-9 on brain-infiltrating myeloid cells (and sham microglia).

Since greater than 80% of strokes occur in individuals over the age of 65, and the elderly have both a higher in-hospital mortality and poorer functional outcomes after an ischemic event [22], it is critical to investigate the therapeutic potential of CD147 inhibition in an aged animal model. 20-month-old male mice underwent 60-minute MCAO (n=7) or sham (n=5) surgery and flow cytometry was performed at 72 hours post-stroke to quantify relative protein expression of CD147 and MMP-9. The gating strategy used is depicted in Figure 6A. Following ischemic stroke, Ly6G^+^ neutrophils were found to increase significantly in the brain (p<0.05, Figure 6B).

Surface expression of CD147 was significantly increased in peripheral monocyte populations found in the ischemic brain when compared to sham brain or their circulating counterparts in blood (Figure 6C). Ly6C^lo^ monocytes within the ischemic brain expressed CD147 at a significantly higher level than resident microglia (p<0.01, Figure 6C). CD147 expression was found on approximately 15% of brain-infiltrating Ly6C^lo^ monocytes (Figure 6C) compared to 60% observed in young stroke mice (Figure 3C). Interestingly, in the aged brain we did not observe an increase in MMP-9 expression in resident microglia (Figure 6F). However, Ly6G^+^ neutrophils demonstrated the highest production level of MMP-9 in the ischemic brain at 72 hours (vs. stroke microglia p<0.01, vs. Ly6C^hi^ p<0.001, vs. Ly6C^lo^ p<0.05, Figure 6F).

**Figure 7:**
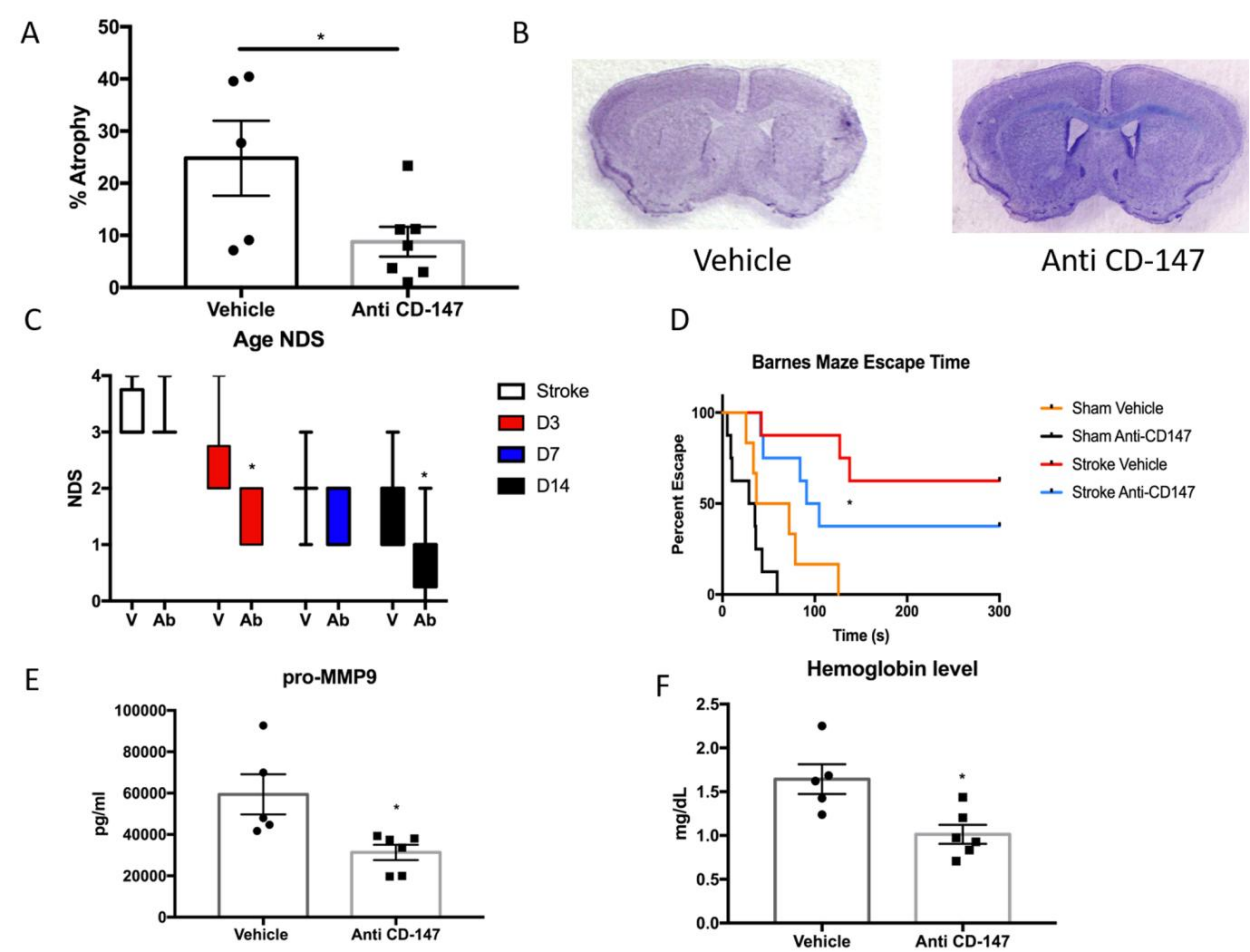
**CD147 blocking antibody reduced infarct volume and improved cognitive outcomes in aged male mice.** (**A**) CD147 blocking antibody administration reduced the amount of atrophy witnessed in the aged brain following stroke compared to IgG control at day 14. (**B**) Representative cresyl violet stained brain slices. (**C**) Neurological deficit scores showed improvement at day 3 and day 14 following stroke. (**D**) Cognitive impairment, as measured by escape time on the Barnes Maze, was prevented in stroke mice that received CD147 blocking antibody. (**E**) Levels of pro-MMP-9 were reduced in stroke mice receiving blocking antibody. (**F**) Brain hemoglobin, a reflection of blood brain barrier breakdown and hemorrhagic transformation was decreased in blocking antibody treated mice.

Anti-CD147 antibody administration reduced brain tissue atrophy (IgG 24.8±7.17 vs. CD147 Ab 8.78±2.86, p<0.05, n=7/grp Figure 7A) at 14 days, representative cresyl violet images shown in Figure 7B. Anti-CD147 treated mice also had lower NDS at 72 hours and at day 14 after stroke compared to vehicle treated stroke mice (p=0.01 and p<0.005 respectively, generalized linear mixed model with Bonferroni correction, Figure 7C). Aged stroke mice receiving anti-CD147 also had improved performance in learning and spatial memory in the Barnes maze compared to mice receiving control IgG (p=0.01, Figure 7D).

As CD147 is an inducer of MMPs, we examined the levels of active MMP-9 in the ischemic brain. CD147 blockade reduced the levels of active MMP-9 compared to vehicle at 72 hours following ischemia (IgG 59400±9693 vs. anti-CD147 31348±3722 pg/ml, p=<0.05, n=5,6 respectively, Figure 7E). Furthermore, upon sacrifice we observed a number of aged animals had evidence of cerebral hemorrhage. Therefore we performed a hemoglobin assay on brain lysates of stroke animals treated with blocking antibody or vehicle. CD147 blockade reduced hemoglobin concentrations in the brain at 72 hours following ischemia (IgG 1.644±0.17 vs. anti-CD147 1.014±0.108 mg/dL, p<0.05, n=5,6 respectively, Figure 7F).

### Serum CD147 is an independent predictor of poor outcomes in human stroke patients

**Figure 8:**
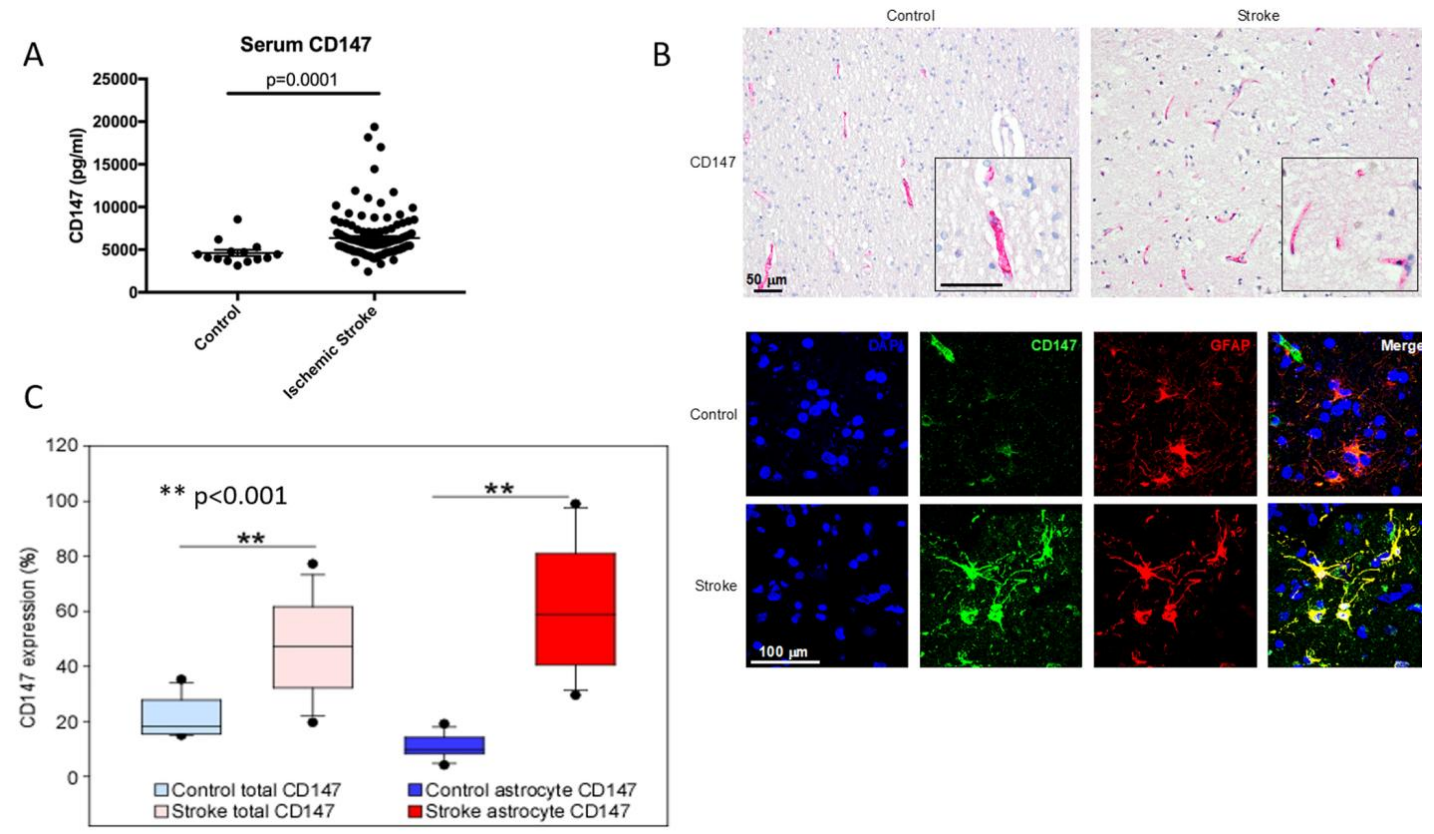
**CD147 is expressed on human astrocytes and levels of CD147 correlate with poor outcomes.** (**A**) Serum levels of human CD147 24 hours post stroke. (**B**) Representative immunohistochemical staining patterns of CD147 in human brain tissues from age-matched control and ischemic stroke subjects (Top, scale bar = 50 μm). Immunofluorescence (IF) staining in human brain tissues (Bottom, scale bar = 100 μm). Simple IF with DAPI (blue), CD147 (green), and GFAP (red). Double IF with CD147 (green) and GFAP (red). (**C**) Quantification of levels of CD147 and GFAP expressing CD147 in human brain tissue.

Given our findings in mice, we then examined CD147 in human stroke patients to determine if CD147 levels correlated with functional outcome after stroke. ELISA was performed on human serum samples obtained at 24 hours after the onset of ischemic stroke and compared to controls. Serum CD147 concentrations were significantly higher in stroke patients (6367±2610 pg/ml) compared to control patients (4625±1369 pg/ml, p<0.0001) 24 hours after symptom onset (Figure 8A). Most importantly, higher serum concentrations of CD147 at 24 hours post-stroke was a significant independent predictor of poor outcomes at 12 months after stroke, even after multivariate adjustment for potential confounding factors, including initial stroke severity (Table 1). In the logistic regression, higher levels of CD147 at 24 hours after stroke was significantly and independently associated with greater likelihood of a negative outcome 12 months after stroke as measured by a Modified Rankin scale (mRS) ≥2 (on a six point scale with 0 representing no residual deficits, to 6 representing death). Higher levels of CD147 were also predictive of outcome on a composite measure defined by death or worse functional outcomes measured by a Modified Barthel Index of ≤ 14 (a scale that ranges from 0-20, with 20 being normal and 0 being death) at 12 months. Patient demographic data is shown in Supplementary Table 1.

**Table 1:** CD147 is an independent predictor of poor outcomes 12 months after stroke.

**Poor Functional Outcome Measure**	**Univariate^a^**	**Multivariate^b^**
	***P* Value**	***P* Value**
Modified Rankin	.111	.343
Score ≥ 2		
*3 months*		
Modified Rankin	.002	.022
Score ≥ 2		
*12 months*		
Modified Barthel	.076	.184
Index ≤ 14 or Death		
*3 months*		
Modified Barthel	.004	.013
Index ≤ 14 or Death		
*12 months*		

^a^Mann-Whitney U-test

^b^Logistic regression analysis, controlling for age, initial stroke severity as | measured by NIH stroke scale, white blood cell counts, intra-arterial therapy, medications (anti-cholesterol medications, anti-hypertensive medications) and common comorbidities (heart disease, atrial fibrillation, diabetes, arthritis).

Lastly, we performed IHC analysis of post-mortem brain sections from human stroke patients to determine the cellular location of CD147 as well as levels of both CD147 and MMP-9 in brain tissue. Overall expression of CD147 and the percentage of astrocytes with CD147 expression was increased in stroke tissue compared to controls (% CD147 expression 20.72±1.80 vs stroke 47.00±4.27, p<0.001 % astrocyte CD147 protein expression control 10.22±1.07 vs. stroke 61.11±5.66, p<0.001, n = 17/grp. Figure 8B and 8C). Patient demographics and characteristics are shown in Table 2. Signal intensity of CD147 and MMP both correlated with hemorrhage size (Tables 3 and 4).

**Table 2:** Demographic and characteristics of human post mortem cases.

**Characteristics**	**Control N=17**	**Stroke N=17**	***P* value**
Age (year)	71.7 ± 15.7	74.3 ± 13.9	0.61^a^
Male, n (%)	10 (58.8)	7 (41.2)	0.31^b^
Hemorrhage	0.0 (0.0 - 0.0)	22.2 (5.6 - 61.1)	<0.001^b^
MMP-9 level	3.3 (2.22 - 5.4)	19.1 (5.1 - 24.3)	0.002^b^
CD147 level	17.2 (15.2 - 27.6)	47.2 (32.2 - 61.7)	<0.001^b^

MMP-9, Matrix metallopeptidase 9

^a^Independent t-test, mean ± standard deviation were reported

^b^Mann-Whitney U-test, median (interquartile range) were reported

**Table 3:** Correlation between different variables in all human post mortem cases.

**Parameters**	**Age**	**Hemorrhage**	**MMP-9**	**CD147**
Age	1			
Hemorrhage	0.452**	1		
MMP-9	0.44**	0.827**	1	
CD147	0.264	0.786**	0.744**	1

MMP-9, Matrix metallopeptidase 9

N=34. Spearman's correlation. ** p<0.01

**Table 4:** Correlation between different variables in the human post mortem cases of acute and sub-acute infarct ages.

**Parameters**	**Age**	**Hemorrhage**	**MMP9**	**CD147**
Age	1			
Hemorrhage	0.556*	1		
MMP-9	0.620**	0.853**	1	
CD147	0.461	0.488*	0.386	1

MMP-9, Matrix metallopeptidase 9

N=17. Spearman's correlation. * p<0.05; ** p<0.01

## DISCUSSION

This study demonstrates several important new findings. First, CD147 expression increases on infiltrating monocytes, and co-localizes with astrocytes and endothelial cells at 72 hours after stroke; secondly, MMP-9 activity is increased in microglia at 72 hours after stroke without concurrent up-regulation of CD147 expression; thirdly, CD147 blockade decreases expression and activity of MMP-9; fourthly, inhibition of CD147 is neuroprotective in both young and aged mice and reduced hemorrhagic transformation in aged mice. Lastly, we found that serum concentrations of CD147 are higher in stroke patients with poor 12-month outcomes, independent of age, initial stroke severity and other co-morbidities. CD147 expression increased in human brain after stroke, which was associated with increased astrocytic CD147 expression, MMP-9 expression, and hemorrhage.

CD147 is an upstream mediator of MMP-9 activity and has been linked to an increased inflammatory response and enhanced leukocyte recruitment in models of experimental autoimmune encephalomyelitis, where inhibition of CD147 blocks leukocyte entry and reduces disease severity [15]. Consistent with the known pattern of peak peripheral leukocyte infiltration after ischemic stroke at 72 hours, the present study showed that CD147 is expressed on astrocytes, endothelial cells and monocytes in the brain at 72 hours after stroke, and may contribute to secondary histological damage via augmented MMP-9 activity, exacerbated BBB damage, and enhanced infiltration of peripheral leukocytes. Administration of a CD147 blocking antibody reduced infarct volumes when measured during the acute phase of stroke, consistent with previous findings [17], while diminishing hemispheric atrophy during the later phase of stroke recovery. Coinciding with reduced atrophy, young mice receiving antibody treatment exhibited improved neurological outcomes and functional activity over the course of the study. Spatial learning and memory in these mice was comparable to sham animals when measured on the Barnes maze test.

MMP-9 levels are strongly associated with BBB dysfunction in both animal and clinical studies [12, 25]. Previous studies have also shown that plasma levels of MMP-9 are higher with age (>60 years) and this may contribute to higher mortality and worse functional outcomes seen in older individuals [26]. Coinciding with these clinical findings, our lab has shown that following focal ischemia aged mice have worse functional outcomes than young animals despite having similar or smaller infarcts [27]. Aged mice receiving CD147 blocking antibody showed a reduction in hemispheric atrophy at 14 days following ischemia. Corresponding with this data, antibody-treated aged mice also demonstrated improved neurological outcomes as well as preserved spatial learning and memory when tested on the Barnes maze. This suggests that inhibition of CD147 may improve functional and cognitive outcomes in elderly populations.

This work also shows that the downstream target of CD147, namely MMP-9, was affected by antibody therapy. Tissue levels of active MMP-9 and hemoglobin were both decreased in the ischemic brains of mice that received anti-CD147 suggesting that the integrity of the BBB is pivotal in regulating secondary injury due to leukocyte migration and subsequent hemorrhagic transformation, variables that predispose to poorer outcomes following stroke [28–32].

Previous studies have shown that CD147 concentrations are elevated in serum of patients with chronic inflammatory conditions [33, 34]. Our study suggests that patients with high serum levels of CD147 at 24 hours after stroke have poorer outcomes even out to 12 months after stroke. Although the risk was small, the fact that this was found in a relatively small cohort of patients at chronic follow up suggests that this is an important signaling pathway and that CD147 levels could be developed into a valuable biomarker of recovery. Future studies using larger cohorts, as well as investigation of CD147 levels in patients with hemorrhagic transformation and intracerebral hemorrhage are needed. One limitation is that stroke size/volume data was not available for this human cohort, as these were brain slices from a brain bank without linked MRI imaging. The chronic role of CD147 in stroke recovery, as well as blood brain barrier dysfunction at 12 months after injury, and potential efficacy in female animals will provide additional insights into the function of CD147 in stroke.

Currently, the treatment of ischemic stroke is limited to tissue plasminogen activator (tPA), which must be administered within 4.5 hours of symptom onset, or mechanical thrombectomy. Thrombectomy is beneficial up to 24 hours following onset of stroke symptoms, but unfortunately, it is only available at a minority of medical centers. [35] Here, we administered two doses of CD147 blocking antibody, the first at reperfusion (60 minutes after the onset of ischemia), and again 24 hours later, mimicking thrombectomy eligible candidates. We observed both a reduction in cerebral atrophy 14 days later and an improvement in cognitive outcomes. Importantly, improvements were also seen in a translationally relevant model, aged animals. As the levels of CD147 remain elevated in stroke patients at 24 hours, and levels of CD147 correlate with poorer outcomes, the translational relevance of delaying the initial dose must be further explored.

Microglia play a significant role in secondary inflammation after ischemic stroke [7]. Microglial expression and activation of MMP-9 is up-regulated via Toll-like receptor 2 signaling [36, 37]. Much evidence supports the function of neutrophils as a critical source of MMP-9 in the microenvironment in tumor models [38] and perhaps more so in stroke. Our study showed that 72 hours after injury both microglia and neutrophils increased expression of MMP-9, yet neither expressed CD147. This suggests that both microglia and neutrophils contribute to MMP-mediated BBB breakdown, potentially facilitating the recruitment of peripheral monocytes to the site of injury likely through astrocyte-mediated up-regulation of CD147. A recent study has shown that while microglia play a role in the initial inflammatory response after stroke, at 72 hours microglia are decreased in number, as they are also vulnerable to ischemic injury [7]. An astrocyte-specific CD147 knockout mouse would further clarify the events leading to microglial up-regulation of MMP-9, BBB permeability and leukocyte extravasation. There was also a dramatic increase of CD147 on infiltrating monocytes and it is possible that up-regulation of CD147 in these cells is the primary inducer of MMP-9 activity in microglia. Both soluble and membrane bound forms CD147 has been shown to induce MMPs. Like other Ig containing molecules, CD147 has been shown display homotypical interactions, although this does not rule out distinct receptors for CD147 [39] Integrins α3β1 and α6β1 have been shown to co-localize with CD147 and play important roles in cell adhesion, chemotaxis and MMP induction [40–42] This may provide insight into a signaling mechanism between the up regulation of CD147 on infiltrating monocytes and the increase of MMP-9 on microglia and neutrophils. Additionally, cyclophilin binding of CD147 plays an important role in chemotaxis, when this interaction was disrupted with an anti-CD147 antibody, decreased levels of cytokines and immune cells were observed therefore greatly decreasing lung inflammation and pathology in asthma [43]. When administered within 24 hours of the onset of ischemia a CD147 blocking antibody reduced the number of brain infiltrating neutrophils, macrophages and both CD4 and CD8 T-cells [17]. These data taken collectively with ours suggest a peripheral role of the blocking antibody but does not rule out the possibility of crossing a permeable blood brain barrier and exerting a central effect. It will be interesting to understand to what effect, if any, delayed administration of anti-CD147 antibody will have on stroke outcomes.

Monocytes have been shown to exacerbate injury in several brain injury models [15, 44, 45]. Ly6C^lo^ monocytes, “patrolling” monocytes [46], found in the brain at 72 hours after stroke had a 40-fold increase in CD147 expression compared to circulating blood monocytes. CD147 expression on Ly6C^hi^ inflammatory monocytes was much lower in the ischemic brain compared to LyC6^lo^ expression, suggesting CD147 plays a role in monocyte recruitment resulting in phenotype switching. The increase in CD147 expression on infiltration peripheral monocytes suggests that changes in CD147 expression occur quickly and could propagate a positive feedback loop, increasing microglial MMP-9 activity and allowing further leukocyte entry. Blocking CD147 reduced damage and whether this is due to reductions in monocyte entry, neutralization of neutrophil-derived MMP-9 activity, or a primary effect on CNS resident cells will require further investigation.

## CONCLUSION

CD147 is a major regulator of the BBB after stroke, facilitating MMP-9 mediated BBB breakdown and recruitment of peripheral leukocytes into the CNS. Brain infiltrating monocytes up-regulate CD147, while resident microglia show increased MMP-9 activity but no up-regulation of CD147 acutely following ischemia. This suggests that the rise in microglial MMP expression is a result of the higher CD147 on astrocytes and infiltrating monocytes. In humans, increased serum CD147 in stroke patients predicted poor long-term outcome. Most importantly, blocking CD147 leads to significantly reduced histological damage while improving functional and cognitive outcomes in both young and aged mice. Long-term effects of CD147 inhibition on chronic re-modeling after stroke is needed, but these studies suggest that CD147 may be a therapeutic target for stroke.

## MATERIALS AND METHODS

### Animals

Male C57BL/6 mice 6-8wks of age were obtained from Charles River (Wilmington, MA, USA), and male C57BL/6 mice 20 months of age were obtained from the National Institute on Aging and allowed to acclimate for a minimum of four weeks before use. All mice were housed in a temperature- and humidity-controlled vivarium, 5 per cage (11”L, 6”W, 6”H) with a 12-hour light/dark schedule with *ad libitum* access to food and water. All animal experimental work was approved by the Institutional Animal Care and Use Committee (IACUC) at University of Connecticut Health Center and at the University of Texas Health Science Center at Houston, and was performed in accordance with National Institutes of Health guidelines.

Five different cohorts of mice were used in this study; young mice were used unless otherwise specified. The first cohort of mice was used for immunohistochemistry and seven mice (*n*=4 stroke; *n*=3 sham) were sacrificed at 72 hours. The second cohort used was for flow cytometry and intracellular staining and ten mice (*n*=5/group) were used. The third cohort, fourteen mice (*n*=7/group), was used to examine the effects of blocking CD147 activity using a function-blocking antibody. 72-hour time-point studies had a mortality rate of 15%. The fourth cohort, 36 mice (*n*=9/group), was used to examine the long-term outcomes of blocking CD147 in young male mice. The fifth cohort, 36 mice (*n*=9/group), was used to examine the long-term effects of blocking CD147 in aged male mice. All experimental numbers were determined by power analysis (G power) to utilize the lowest number of mice, mice were assigned randomly into treatment groups using a computer based model (SPSS) and all assays and behavioral tests were performed by a blinded investigator.

### Middle cerebral artery occlusion model

Focal transient cerebral ischemia was induced by 60 minutes of reversible unilateral middle cerebral artery occlusion (MCAO) under isoflurane anesthesia followed by reperfusion as described previously [47–49]. Sham animals underwent the same procedure but the suture was not advanced into the MCA. During surgery and ischemia, rectal temperature was monitored with a Monotherm system (V WR LabShop, Batavia, IL, USA) and maintained at approximately 37 °C. Cerebral blood flow reduction of >80% of baseline after suture insertion was confirmed in all stroke animals by Laser Doppler Flowmetry (Moor Instruments).

### Protein extraction

Ipsilateral hemispheres were lysed in cold 2x RIPA buffer (Cell Signaling) using Dounce homogenizers. The lysate was spun at 800g for 10 minutes at 4°C and sonicated for 10 seconds three times as described in Li [48]. Aliquots were taken for western analysis.

### Western analysis

30 μg of protein taken from whole cell lysate samples were run on 12% TGX Criterion gels and transferred to a polyvinylidene diflouride membrane. All blots were blocked with 5% bovine serum albumin (BSA, Sigma-Aldrich) and incubated overnight with rabbit anti MMP-9 (Abcam, 1:1000) and mouse anti-actin (Sigma-Aldrich 1:5000) in TBS with 4% BSA and 0.1% TWEEN. Secondary antibodies (anti-rabbit IgG 1:5000 for MMP-9, anti-mouse 1:10,000 for actin) (GE Healthcare UK Limited) were diluted, and ECL detection kit (Amersham Biosciences) was used for signal detection. Densitometry was performed using ImageJ (NIH) as previously described in Li [48].

### Histology

Mice were anesthetized with 0.1mL/10g body weight dose of avertin (Sigma) dissolved in 2-Methyl-2-Butanol and then transcardially perfused using ice-cold sodium phosphate-buffered saline (PBS) followed by 4% paraformaldehyde. Brains were extracted and post-fixed for 24h in paraformaldehyde and then transferred to a 30% sucrose solution and sectioned. These sections were stored in antifreeze at -20°C slide-mounted and used for immunohistochemistry or stained with cresyl violet for infarct analysis as described in Venna [49].

### Immunohistochemistry

Thirty-micron sections were slide mounted and incubated in Normal Goat Serum (Sigma) with 0.15% TritonX in PBS as previously described [50]. Sections were incubated overnight with rat anti-CD147 (1:200, ABDserotec), and then incubated for 60 minutes with anti-host antibody conjugated with a fluorophore (1:1000), DAPI nuclear stain solution (1:1000; Invitrogen), and GFAP primary conjugated antibody (1:200, Invitrogen) as previously described in [51]. Images were then taken using an inverted light Zeiss-Axiovert fluorescence microscope [51]. Quantification was performed in pre-specified regions of the ipsilateral cortical region (approximately 3 mm laterally and 2 mm ventrally from midsagittal line) by a blinded investigator. Pearson’s correlation coefficient was determined using Image J software. Regions of interest were drawn around cells. Values range from 0 to 1 (0 - no co-localization; 1 - all pixels co-localize). A two-tailed student’s t-test was used to determine significance.

### Flow cytometry

Mice were perfused with 60 mL of cold, sterile PBS and stroke-side brain hemispheres were placed in complete RPMI 1640 (Lonza) medium and mechanically and enzymatically digested in collagenase/dispase (1 mg/mL) and DNAse (10mg/mL; both Roche Diagnostics) for 1hr at 37°C. The cell suspension was filtered through a 70μm filter. Leukocytes were harvested from the interphase of a 70%/30% Percoll gradient. Blood was collected by cardiac puncture with a heparinized syringe. Red blood cells were lysed following three consecutive 8-minute incubations with tris ammonium chloride (StemCell Technologies) on ice. Brain and blood cells were washed and blocked with mouse Fc Block (93) prior to staining with primary antibody-conjugated flourophores: CD45-eF450 (30-F11), CD11b-APCeF780 (M1/70), Ly6G-FITC (1A8), Ly6C-PerCPCy5.5 (HK1.4), CD3e-APC (145-2C11), and CD147-PE (RL-73). All antibodies were purchased from eBioscience. For live/dead discrimination, a fixable viability dye, carboxylic acid succinimidyl ester (CASE-AF350, Invitrogen), was diluted at 1:300 from a working stock of 0.3mg/mL. Cells were briefly fixed in 2% paraformaldehyde (PFA). Data were acquired on a LSRII using FACsDiva 6.0 (BD Biosciences) and analyzed using FlowJo (Treestar Inc.). Fluorescence minus one (FMO) controls were used to determine the positivity of each antibody.

For intracellular cytokine staining, an *in vivo* brefeldin A (BFA) protocol was followed. Briefly, 10mL/kg of BFA (Sigma, 0.5mg/mL in DMSO) was intraperitoneal injected. Ten hours later, animals were sacrificed and tissue was harvested as noted above. Prior to staining, 1 μl of GolgiPlug containing BFA (BD Biosciences) was added to 800 μl complete RPMI and cells were incubated for 2h at 37C (5% CO2). Afterward, cells were re-suspended in Fc Block, stained for surface antigens and washed in 100 μl of fixation/permeabilization solution (BD Biosciences) for 20 minutes. Leukocytes were then washed twice in 300μl permeabilization/wash buffer (BD Biosciences) and resuspended in an intracellular antibody cocktail containing MMP-9-PE (S51-82; StressMarq Biosciences, Inc.) and subsequently fixed (N=4 SH and 6 ST) [7].

### CD147-blocking antibody

Anti-CD147 (eBioscience) or rat-IgG control (100 μL of antibody at a 0.1mg/ml dilution) in ultra-pure water was administered by tail-vein injection. Treatments were given at reperfusion with an additional dose 24 hours after stroke. Mice were assessed for NDS and then sacrificed at 72 hours for 2,3,5-triphenyltetrazolium chloride (TTC) staining as described in Li [48]. Separate cohorts of young and aged mice were used for 14-day survival studies following the same dosing protocol. Animals were randomly assigned to treatment groups, all histological and behavioral analyses were performed by a blinded observer.

### 2,3,5-triphenyltetrazolium chloride (TTC) staining

Brains from antibody and vehicle treated mice were extracted after euthanasia and cut into five 2-mm coronal sections, and stained with 1.5% 2,3,5-triphenyltetrazolium chloride (TTC) as in Venna [49]. Infarct volumes were analyzed by an investigator blinded to treatment group using Sigma Scan Pro (San Jose, CA, USA) as in Venna [49].

### Neurological deficit score (NDS)

Mice were given a neurological deficit score (NDS) as described in [51]. Neurological deficits were scored using the following rubric on a scale from 0-5: 0 = no deficit, 1 = forelimb weakness and torso turning to ipsilateral side when held by the tail, 2 = circling to affected side; 3 = unable to bear weight on affected side and circling immediately when placed on a bench, 4 = no spontaneous locomotor activity or barrel rolling, and 5 = dead. All studies were performed blinded to treatment.

### Corner test

This test detects integrated sensory-motor function as it involves stimulation of vibrissae sensory) and rearing (motor). Testing was carried out described in Li et al. [52], briefly, a mouse is encouraged to enter a 30-degree corner created by two cardboard pieces. Once in the corner, the boards stimulate both sides of the vibrissae, the mouse then rears forward and up, turning to face the open end. Twenty trials are performed and the percentage of right turns is calculated.

### Barnes maze

Barnes maze was conducted on an elevated circular platform (92 cm diameter) with 20 evenly spaced holes (5 cm diameter). A randomly chosen hole was designated as the escape hole, which allowed the animal to escape the platform into a dark rectangular box below. During training trials, mice learn the location of the escape hole by spatial clues positioned around the platform. All training trials and the test trial were performed in a dark room with the platform lit by bright white light. Animals received 3 training trials followed by a test trial 4 hours later on day 14-post ischemia. During the first training trial, the mouse was placed into the center of the arena and then guided to the escape hole by a clear cylindrical chamber, which it was allowed to explore for 1 minute. During the second and third trials the animal was again placed into the center of the platform and allowed to freely explore the arena for 5 minutes. At the end of each trial if the animal did not find the escape hole it was guided to it using the same clear chamber. The arena was cleaned between trials. The testing period consisted of one five-minute trial. The trial was terminated when the animal entered the escape hole or at the end of 5 minute period. Any animal that did not find the escape hole once during any training trial was excluded. A camera was mounted above the maze to monitor performance through a video tracking system (Noldus EthoVision XT). All testing was performed blinded to treatment group.

### Human studies

Samples were collected at a certified JCO Comprehensive Stroke Center. Blood draws occurred at 24 +/- 6 hours after the onset of ischemic stroke and blood was allowed to clot at room temperature before centrifugation and removal of the serum layer. Ischemic stroke patients selected for this study were identified as having acute-onset, focal neurological deficits with positive evidence of cerebral infarction by CT or MRI. Patients with autoimmune disease and active cancer were excluded. The primary outcome measure was poor functional outcome as measured by a Modified Rankin scale (mRS) ≥ 2 (on a six point scale with 0 representing no residual deficits, to 6 representing death) and by a composite measure of negative outcome defined by Modified Barthel Index (MBI) of ≤ 14 (a scale that ranges from 0-20, with 20 being normal) or death at 3 and 12 months. The mRS is used to measure overall disability and independent, while MBI measures physical disability and the performance of activities of daily living. Univariate analysis between CD147 levels at 24 hours and poor composite outcome was conducted by Mann-Whitney U Test. In order to determine whether CD147 level was an independent predictor of poor outcome, a multivariate analysis was constructed to control for potential confounding factors including age, initial stroke severity as measured by NIH stroke scale, white blood cell counts, intra-arterial therapy, medications (anti-cholesterol medications, anti-hypertensive medications) and common comorbidities (heart disease, atrial fibrillation, diabetes, arthritis). For patient demographics, see Supplementary Table 1. For these studies, the criterion of statistical significance was set at 0.05. All analysis was performed using SPSS (Statistical Package for the Social Science) v21.

Immunohistochemistry analyses for human brain sections were performed as described previously [53]. Anti-MMP-9, SMC-396D, 1:250 and anti-CD147, LS-B12190, 1:100 as primary antibodies and anti-mouse-alkaline phosphatase (AP) as secondary antibody were used to examine the total 34 brain samples. Subsequently, the primary antibodies (anti-CD147, LS-B12190, 1:100 and anti-GFAP, ab53554, 1:1000) and secondary antibodies (anti-rabbit 488 and anti-goat 594, 1:100) were used to perform double labeling immunofluorescence analyses. The images were collected using a Leica confocal microscope (Leica, Heerbrugg, Switzerland). All slides were analyzed using ImageJ (Colocalization colormap plugin) [54] by an investigator blinded to case demographics and stroke condition. Fluorescence expression of the CD147 and GFAP signals measured in co-localizing and not co-localizing ROIs, selected on the color maps above. Data are expressed as mean values ± SEM; n=34 ROIs for each condition on images obtained from three independent experiments. Statistical significance is calculated using Mann-Whitney U-test; **P<0.001.

### ELISA

Human serum samples were run as per the manufacturer’s instructions on Human anti-CD147 ELISA (R&D Systems).

### Statistical analysis

Statistics are presented as means ± SEM for all experiments. Statistics were performed using GraphPad Prism. A student t-test was performed when comparing two groups and ANOVA with repeated measures for groups with multiple comparisons with post-hoc correction. A Mann-Whitney U test was used for the ordinal NDS. A probability value of p < 0.05 was considered statistically significant. Cox regression model was used to compare the time to find the exit in the Barnes maze test among different groups, adjusted for multiple testing by Tukey's method. Generalized linear mixed model was used to model the repeatedly measured ordinal variable NDS on the time and groups with multinomial distribution and cumlogit link function. Group comparisons were also performed at each time point, adjusted for multiple testing by Bonferroni correction.

For human samples, univariate analyses were done to explore CD147 levels in groups defined by patient and disease related characteristics including: sex, age, and comorbidities. Groups were also defined by outcomes, such as mortality, NIH stroke scale at discharge, and functional outcomes at 12 months post stroke using the Barthel and Modified Rankin scales. Comparisons were run based on using Wilcoxon Ranked Sum Test or Kruskal-Wallis (when more than two groups were defined) tests within using SPSSv21. Following these analyses, a multivariate approach using logistic regression was used to predict each of the outcome measures with CD147 level as the key predictor of interest and all factors associated with CD147 level (or the outcomes) as covariates.

## Supplementary Material

Supplementary Table 1
