# Supplementary material for: EMMPRIN/CD147 plays a detrimental role in clinical and experimental ischemic stroke
Source: Aging (Albany NY). 2020 Mar 19;12(6):5121–39. doi: 10.18632/aging.102935 (PMC7138568; doi:10.18632/aging.102935)
Supplement: Supplementary Table 1 [file aging-12-102935-s001..pdf]

SUPPLEMENTARY TABLE

Supplementary Table 1. Demographics and comorbidities of human post mortem stroke cases.

Variable	Measure
Age (yrs)	67 (58.7-80.2)
Gender (male)	74 (58.9%)
Heart Disease	42 (33.1%)
Arial Fibrillation	30 (23.1%)
Diabetes Mellitus	45 (34.6%)
High Cholesterol	91 (70.0%)
Hypertension	104 (80.0%)
Smoking	26 (20%)
Arthritis	13 (10%)
**Subtype:**	
Large vessel	9 (17.65%)
Cardioembolic	17 (33.33%)
Small vessel	19 (37.25%)
**Stroke Severity (NIHSS)**	
Mild: ≤4	70 (53.8%)
Moderate: ≥5 ≤12	30 (23.1%)
Severe: ≥13	30 (23.1%)
